# Lung dendritic cells facilitate extrapulmonary bacterial dissemination during pneumococcal pneumonia

**DOI:** 10.3389/fcimb.2013.00021

**Published:** 2013-06-21

**Authors:** Alva Rosendahl, Simone Bergmann, Sven Hammerschmidt, Oliver Goldmann, Eva Medina

**Affiliations:** ^1^Infection Immunology Research Group, Department of Medical Microbiology, Helmholtz Centre for Infection ResearchBraunschweig, Germany; ^2^Institute for Microbiology, Department of Cellular Infection Biology, Technische UniversitätBraunschweig, Germany; ^3^Department Genetics of Microorganisms, Interfaculty Institute for Genetics and Functional Genomics, Ernst Moritz University of GreifswaldGreifswald, Germany

**Keywords:** *Streptococcus pneumoniae*, bacterial dissemination, respiratory infection, dendritic cells, pneumonia, MMP-9

## Abstract

*Streptococcus pneumoniae* is a leading cause of bacterial pneumonia worldwide. Given the critical role of dendritic cells (DCs) in regulating and modulating the immune response to pathogens, we investigated here the role of DCs in *S. pneumoniae* lung infections. Using a well-established transgenic mouse line which allows the conditional transient depletion of DCs, we showed that ablation of DCs resulted in enhanced resistance to intranasal challenge with *S. pneumoniae*. DCs-depleted mice exhibited delayed bacterial systemic dissemination, significantly reduced bacterial loads in the infected organs and lower levels of serum inflammatory mediators than non-depleted animals. The increased resistance of DCs-depleted mice to *S. pneumoniae* was associated with a better capacity to restrict pneumococci extrapulmonary dissemination. Furthermore, we demonstrated that *S. pneumoniae* disseminated from the lungs into the regional lymph nodes in a cell-independent manner and that this direct way of dissemination was much more efficient in the presence of DCs. We also provide evidence that *S. pneumoniae* induces expression and activation of matrix metalloproteinase-9 (MMP-9) in cultured bone marrow-derived DCs. MMP-9 is a protease involved in the breakdown of extracellular matrix proteins and is critical for DC trafficking across extracellular matrix and basement membranes during the migration from the periphery to the lymph nodes. MMP-9 was also significantly up-regulated in the lungs of mice after intranasal infection with *S. pneumoniae*. Notably, the expression levels of MMP-9 in the infected lungs were significantly decreased after depletion of DCs suggesting the involvement of DCs in MMP-9 production during pneumococcal pneumonia. Thus, we propose that *S. pneumoniae* can exploit the DC-derived proteolysis to open tissue barriers thereby facilitating its own dissemination from the local site of infection.

## Introduction

The respiratory tract is a major portal of entry for airborne pathogens. The most common cause of respiratory tract infections worldwide is *Streptococcus pneumoniae* (van der Poll and Opal, 2009). Colonization of the human nasopharynx is the first step in the interaction between *S. pneumoniae* and the human host (Kadioglu and Andrew, 2004). From this location, the bacterium can spread to the lungs causing pneumonia, or further disseminate systemically causing invasive diseases. The first line of host defense against *S. pneumoniae* in the lungs involves resident alveolar macrophages and recruited neutrophils (Dockrell et al., 2003; Hahn et al., 2011). An additional immune cell population residing in the lungs is the dendritic cells (DCs). DCs are potent antigen-presenting cells that play a critical role in the induction of antigen-specific immune responses (Banchereau and Steinman, 1998; Banchereau et al., 2000). In the periphery, DCs have an immature phenotype characterized by high endocytic activity and low T-cell stimulation potential (Banchereau et al., 2000). After pathogen recognition, DCs undergo a coordinated maturation program including the up-regulation of costimulatory molecules (CD40, CD80, and CD86) and MHC Class II, the chemokine receptor CCR7, and the production of proinflammatory cytokines (TNF-α, IL-12, and IL-6) (Banchereau et al., 2000). Mature DCs migrate via the afferent lymphatic vessels into the draining mediastinal lymph nodes, where they can activate antigen-specific T lymphocytes (Banchereau et al., 2000). In the lungs, DCs form an extensive network in close proximity to the respiratory epithelial cells, where they function as immune sentinels for sampling incoming pathogens (Lambrecht et al., 2001; Vermaelen and Pauwels, 2005). After pathogen encounter, airway DCs undergo maturation and rapidly migrate to the T cell area of the draining mediastinal lymph nodes (Xia et al., 1995). Pulmonary DCs have been reported to play an important role in host defense against respiratory pathogens including *Legionella pnemophila* (Ang et al., 2010), *Bordetella pertussis* (Dunne et al., 2009), *Cryptococus neoformans* (Osterholzer et al., 2009), and syncytial virus (Smit et al., 2006). Despite the clinical relevance of pneumococcal pneumonia, scarce information is available regarding the role played by DCs during this infection. *In vitro* studies using low encapsulated or capsule-deficient strains of *S. pneumoniae* have shown that pneumococcal pneumolysin inhibits human DC maturation (Littmann et al., 2009). In addition, Noske et al. (2009) reported that expression of the pneumococcal adherence and virulence factor A (PavA) protected *S. pneumoniae* against recognition and phagocytosis by human DCs. The role of DCs during *S. pneumoniae in vivo* infection has, however, not been investigated so far. In spite of the fact that experimental murine models cannot fully mimic the complexity of human pneumococcal infections, there are far more similarities than differences when comparing pneumonia in humans and pneumonia in mice (Chiavolini et al., 2008). For example, the accumulation of purulent exudate containing neutrophils in the lungs takes place in both human and murine pneumonia (Chiavolini et al., 2008). Furthermore, murine models have been pivotal in the study of pneumococcal pathogenesis and they have the potential to further direct future medical approaches to affected patients.

In this study, we used bone marrow chimeras generated from CD11c-DTR transgenic mice, which allows transient ablation of DCs by administration of consecutive doses of diphtheria toxin (DT), to define the specific role played by pulmonary DCs during pneumococcal pneumonia. Strikingly, our results demonstrated that depletion of DCs improved the resistance of mice to *S. pneumoniae* infection. Furthermore, the beneficial effect afforded by depletion of DCs was due to a significant reduction of *S. pneumoniae* dissemination from the lungs to lymph nodes and systemic tissue. These results provide strong evidence that *S. pneumoniae* might exploit the capacity of DCs to breakdown host barriers to facilitate its dissemination from the local site of infection.

## Materials and methods

### Ethics statement

Animal experiments were performed in strict accordance with the European Health Law of the Federation of Laboratory Animal Science Associations (FELASA). All experiments were approved by the ethical board Niedersächsisches Landesamt für Verbraucherschutz und Lebensmittelsicherheit, Oldenburg in Germany (Permit No. 33.11.42502-04-118/08).

### Bacterial strains and growth conditions

The *S. pneumoniae* strains D39 serotype 2 and *S. pneumoniae* serotype 4, strain TIGR4 were grown routinely at 37°C in Todd-Hewitt broth (Sigma-Aldrich, Munich, Germany) supplemented with 1% (w/v) yeast extract (Sigma-Aldrich) and 10% of heat-inactivated FCS (GIBCO/Invitrogen, Eggenstein-Leopoldshafen, Germany). Bacteria were grown to the Mid-Log phase (OD_600_), collected by centrifugation for 10 min at 4000 rpm, and washed twice with sterile PBS.

### Mice and infection model

**Figure 1:**
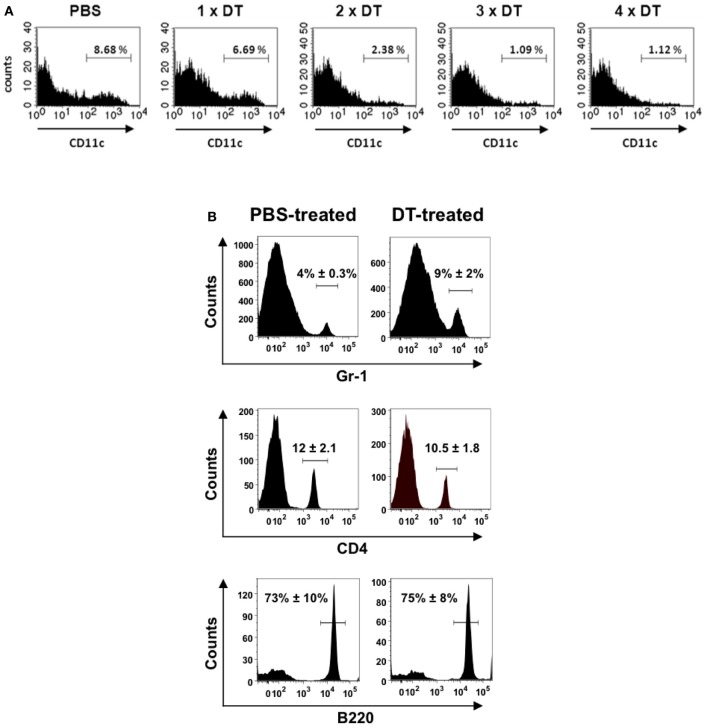
**Depletion of CD11c+ cells in the lungs of CD11c-DTR chimera mice after treatment with successive doses of DT**. CD11c-DTR mice were injected with 1, 2, 3, or 4 successive doses of DT (8 ng/g body weight) or PBS. Lungs were digested and transformed in single cell suspensions 24 h after treatment and stained with PE-conjugated anti-CD11c antibodies for FACS analysis. **(A)** Representative histogram of CD11c expression in lung cells of PBS-treated or DT-treated CD11c-DTR mice. **(B)** Percentage of neutrophils (Gr-1+) (upper histograms), CD4+ lymphocytes (middle histograms) and B cells (B220+) (lower histograms) in the lungs of PBS-treated (left) or DT-treated (right) CD11c-DTR mice. The mean ± *SD* percentages of positive cells are indicated in each histogram. Data shown are representative of one out of three separate experiments.

BALB/c mice were purchased from Harlan-Winkelmann (Borchen, Germany), B6.FVB-Tg [Itgax-DTR/GFP] 57Lan/J (referred to as CD11c-DTR) transgenic mice (Jung et al., 2002) were obtained from Steffen Jung (The Weizmann Institute of Science, Rehovot, Israel). These transgenic mice express the simian DT receptor on the CD11c promoter region, which allows for selective depletion of DCs following the administration of DT. To avoid the lethal effect observed after repeated DT administration, bone marrow chimera mice were generated as previously described (Bar-On and Jung, 2010). Briefly, 2.5 × 10^5^ bone marrow cells from a CD11c-DTR donor were transferred intravenously into BALB/c mice, which were lethally irradiated (950 rads) 24 h earlier. The chimeric animals (referred to as CD11c-DTR chimeras) were treated for 1 week with 0.04% Baytril together with 0.054% glucose in their drinking water. The CD11c-DTR chimeras were ready for experimental use at 8 weeks post-reconstitution. For continuous depletion of DCs, CD11c-DTR chimeras received a daily intraperitoneal injection of DT (Sigma-Aldrich) in PBS (8 ng/g body weight) starting 2 days before infection (Figure 1A). Treatment with DT did not affect the amounts of B or T lymphocytes (Figure 1B). However, the amount of neutrophils (Gr-1+ cells) was significantly increased in the lungs of mice undergoing DT treatment (Figure 1B).

For intranasal infection with *S. pneumoniae*, mice were anesthetized by intraperitonal injection with ketamine (70 mg/kg; Ketavet; Pharmacia & Upjohn, Erlangen, Germany) and xylazine (Rompun® 2%, 20 mg/kg; Bayer, Leverkusen, Germany) and inoculated intranasally with 1 × 10^8^ CFU of *S. pneumoniae*.

### Flow cytometry analysis

Cells were incubated for 5 min with anti-CD32 antibodies to block Fc receptors and then stained with PE-conjugated anti-CD11c, APC-conjugated anti-CD11b, FITC-conjugated anti-CD4, PE-conjugated anti-Gr-1 or PE-conjugated anti-B220 antibodies along with their isotype control antibodies and incubated for 30 min at 4°C. Labeled cells were analyzed by flow cytometry in a FACSCalibur™ (Becton Dickinson).

### Generation of bone marrow-derived dendritic cells

Bone marrow cells were flushed from murine femurs and tibias and cultured for 6 days at 37°C in RPMI 1640 (Invitrogen, Paisley, UK) 5% FCS, 1% penicillin/streptomycin, 50 ng/ml of recombinant mouse GM-CSF (granulocyte-macrophage colony-stimulating factor; Miltenyi Biotech), and 10 ng/ml IL-4 (R&D Systems, Wiesbaden-Nordenstadt, Germany). DCs were gently washed and fed with fresh medium supplemented with GM-CSF (25 ng/ml) and IL-4 (5 ng/ml) on days 2 and 4. On day 6, the DC fraction was enriched using an OptiPrep™ (Axis-Shield, Oslo, Norway) gradient. The purity of the resulting cell population consisted of >80% of DCs as determined by flow cytometry analysis using anti-mouse CD11c-PE antibodies (BD Pharmingen).

### Cytokines and chemokines detection

Cytokines and chemokines were analyzed by multiplex bead technology (Bio-Rad Inc., Hercules, CA, USA) according to the manufacturer's protocol.

### Isolation and phenotypic characterization of lung cells

Lungs were enzymatically digested with 1 mg/ml of collagenase F (Sigma-Aldrich) and 50 U of DNase I (Sigma-Aldrich) in 500 μl RPMI 1640 at 37°C for 30 min. The resulting cell suspension was filtered through a 100-μm strainer, resuspended in RPMI and prepared for further analysis.

### Gentamicin protection assay

Mediastinal lymph nodes were transformed into a single cell suspension, centrifuged at 900 rpm for 8 min and the supernatant was plated in 10-fold serial dilutions onto blood agar plates to determine the number of extracellular bacteria. The cell pellet was then incubated for 2 h in medium containing 100 μg/ml of gentamicin to eliminate extracellular microorganisms and collected by centrifugation. The cell pellet was washed, disrupted after treatment with 50 μl ddH_2_O and the amount of viable intracellular bacteria determined after serial plating onto blood agar.

### DC viability test

Viability of infected DCs was determined using an FITC annexin V apoptosis detection kit I (BD Biosciences) according to the manufacturer's instructions and analyzed by flow cytometry.

### Double depletion of DCs and neutrophils

CD11c-DTR chimeras were injected daily with DT intraperitoneally starting 2 days before infection and intravenously injected with 100 μg of anti-mouse RB6 antibodies 1 day before bacterial inoculation. Control mice received equivalent amounts of isotype control antibodies in sterile PBS.

### Immunofluorescence microscopy

For double immunofluorescence of extracellular/intracellular bacteria, DCs were seeded in sterile coverslips and infected with *S. pneumoniae* for 2 h at a MOI of 20 bacteria per DC. Coverslips were then rinsed to remove unbound cells, and adherent cells were fixed with 3.7% formaldehyde. For double immunofluorescence staining, extracellular bacteria were stained with polyclonal rabbit anti-*S. pneumoniae* antibodies, followed by Alexa green-conjugated goat anti-rabbit antibodies (Sigma-Aldrich, Germany). After several washes, cells were permeabilized by 0.025% Triton X-100 in PBS, washed again, and intracellular bacteria were stained by rabbit anti-*S. pneumoniae* antibodies, followed by Alexa red-conjugated goat anti-rabbit antibodies (Sigma-Aldrich). The fluorescence images were obtained with a confocal laser scan microscope (BIO-RAD, Hercules, CA, USA). Infected DCs showed no significant decrease in cell viability or evidence of apoptosis (data not shown).

### Analysis of mRNA expression

Total RNA was extracted using the RNeasy Kit (Qiagen, Hilden, Germany) according to the manufacturer's recommendations. PCR amplification was performed using a LightCycler 480 Real Time PCR system (Roche Applied Science, Mannheim, Germany) and Maxima SYBR Green qPCR Master Mix (Fermentas, St. Leo-Rot, Germany). The sequence for MMP-9 sense primer was: 5′-GGGAAGGCTCTGCTGTTCAGC-3′, and for the antisense primer: 5′-TCTAGAGACTTGCACTGCACG-3′. The sequence for the ß-actin sense primer was: 5′-TGGAATCCTGTGGCATCCATGAAA-3′ and the antisense primer: 5′-TAAAACGCAGCTCAGTAACAGTCCG-3′. Cycle threshold values for *mmp-9* were normalized to the housekeeping gene β-*actin*. The data were calculated using the Pfaffl equation (Pfaffl, 2001) and expressed as a ratio of the relative mRNA expression in infected samples as compared to that of the uninfected controls.

### Gelatin gel zymography

Culture supernatant of uninfected and *S. pneumoniae*-infected DCs at 24 h postinfection were mixed with equal volumes of 2 × SDS sample buffer containing 10% glycerol, 2% SDS, 0.00125% bromophenol blue and 0.06 M Tris (pH 6.8) and electrophoresed in 10% SDS-PAGE containing 0.1% (w/v) bovine gelatin. Gels were incubated in renaturing buffer (2.5% Triton X-100) at room temperature for 20 min and then incubated for at least 72 h at 37°C in a buffer containing 5 mM CaCl_2_, 0.2 M NaCl, 0.02% (w/v) Brij-35, and 50 mM Tris (pH 7.6). Thereafter, gels were stained for 1 h with Coomassie Dye containing 0.06% Coomassie Brilliant Dye G, 25% (v/v) methanol, 0.005% acidic acid, and 20 mM EDTA. After destaining with 10% acidic acid, gels were imaged on a Cano Scan 9000F.

### *In vivo* vascular permeability assay

Bone marrow-derived DCs were infected with *S. pneumoniae* of 20 bacteria per DC for 90 min. DCs were then washed and further incubated in the presence of antibiotics (100 μg/ml gentamicin, 10 μg/ml penicillin) at 37°C and 5% CO_2_. The culture supernatant was collected after 24 h of culture and stored at −80°C until use. Medium from uninfected DCs as well as medium where only bacteria were added were used as controls. Evans blue (30 mg/kg body weight) was injected intravenously into BALB/c mice. Fifty microliters of the supernatant were injected intradermally into the dorsal skin of anesthetized shaved mice. One hour later the mice were sacrificed and Evans blue was extracted by incubation of the tissue in 500 μl formamide at 60°C for 48 h. The vascular leakage activity was then determined by quantitatively measuring the extracted Evans blue by spectrophotometry at 620 nm using the Sunrise absorbance reader and Magellan™3 software.

### Statistical analysis

Data was analyzed using GraphPad Prism 4.0 (GraphPad software). Comparison between groups was performed by the use of non-parametric Mann–Whitney *U*-test. *P*-values ≤ 0.05 were considered as significant.

## Results

### Depletion of DCs results in enhanced resistance to *S. pneumoniae* infection

**Figure 2:**
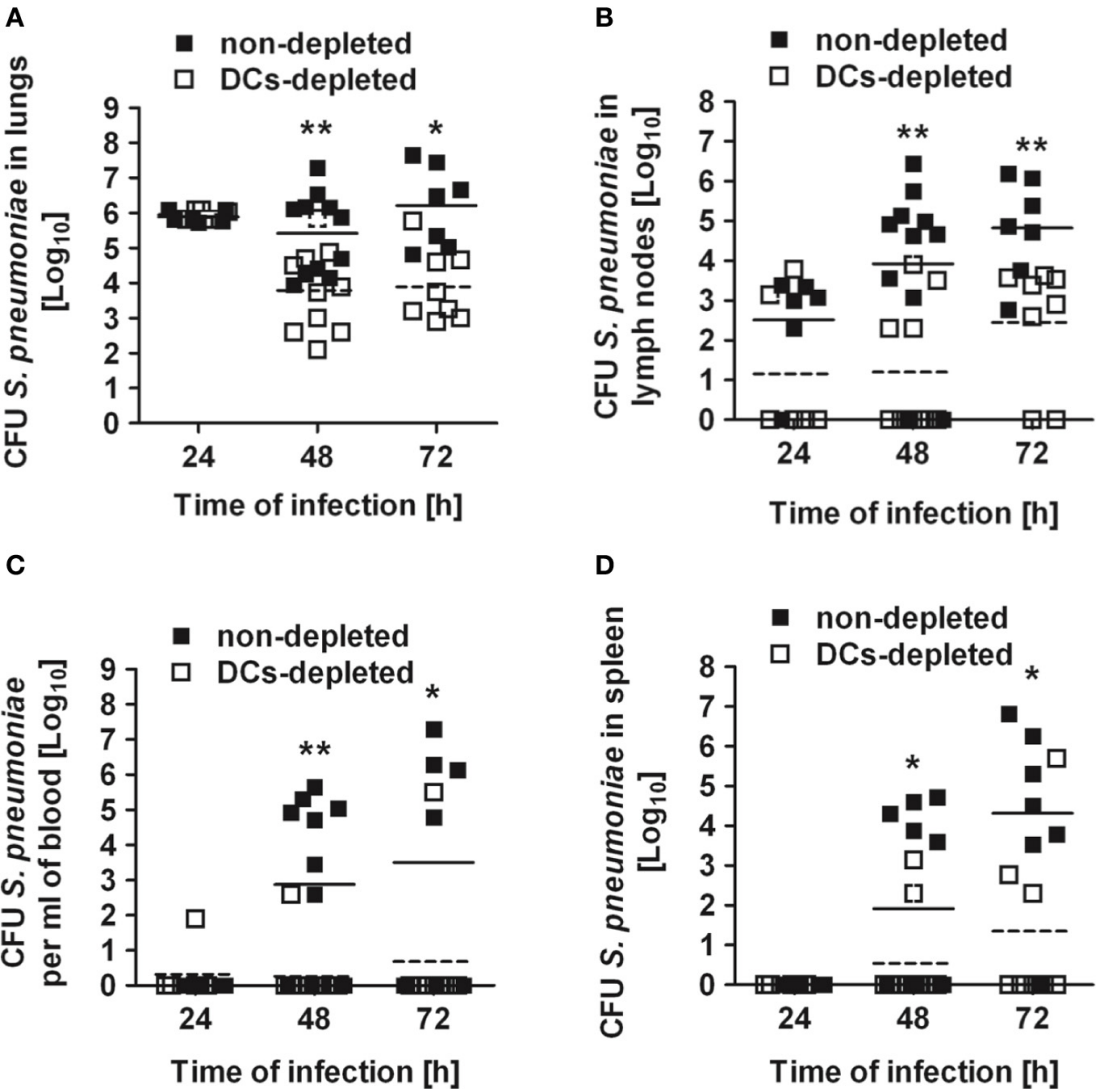
**Bacterial burdens in the organs of DCs-depleted and non-depleted mice after respiratory challenge with *S. pneumoniae***. DCs-depleted (white symbols) and non-depleted (black symbols) CD11c-DTR chimera mice were intranasally inoculated with 1 × 10^8^ CFU of *S. pneumoniae* D39 and the bacterial loads determined in the lungs **(A)**, mediastinal lymph nodes **(B)**, blood **(C)**, and spleen **(D)** at 24, 48, and 72 h after bacterial inoculation. Each symbol represents an individual animal. Horizontal continuous lines indicate the mean value for non-depleted mice and horizontal broken lines indicate the mean value for DCs-depleted mice. One experiment out of three is shown. ^*^p < 0.05 and ^**^p < 0.01.

To determine the relevance of DCs for the control of *S. pneumoniae*, CD11c-DTR chimera mice were depleted of DCs and intranasally infected with a sublethal inoculum of *S. pneumoniae* strain D39 (1 × 10^8^ CFU). During the first 24 h following infection, the numbers of bacteria in the lungs recovered from both DCs-depleted and non-depleted mice were nearly identical (Figure 2A). Extrapulmonary pneumococci appeared in the draining mediastinal lymph nodes by 24 h of infection in 80% of the non-depleted mice but only in 20% of DCs-depleted animals (Figure 2B), indicating that bacteria dissemination from the lungs was more efficient in the presence of DCs. Dissemination of *S. pneumoniae* to the draining lymph nodes was followed after 48 h by systemic dissemination to blood (Figure 2C) and peripheral organs such as spleen (Figure 2D). Again, this dissemination occurred to a significantly higher extent in non-depleted mice. By 48 h and 72 h of infection, the amount of pneumococci in all organs was significantly lower in DCs-depleted than in non-depleted mice. Furthermore, non-depleted mice demonstrated more severe signs of morbidity characterized by lethargy, ruffled fur and hunchbacked posture after *S. pneumoniae* challenge than DCs-depleted animals.

The superior resistance of DCs-depleted mice to *S. pneumoniae* was not strain-specific since similar results were obtained with *S. pneumoniae* strain TIGR4 (see Figure S1 in supplementary material). We also excluded a potential effect of the DT treatment *per se* by demonstrating that wild-type BALB/c mice treated with DT exhibited a similar infection kinetic than untreated animals (see Figure S2 in supplementary material).

### DCs-depleted mice exhibit lower levels of systemic inflammation after intranasal inoculation with *S. pneumoniae* than non-depleted mice

**Figure 3:**
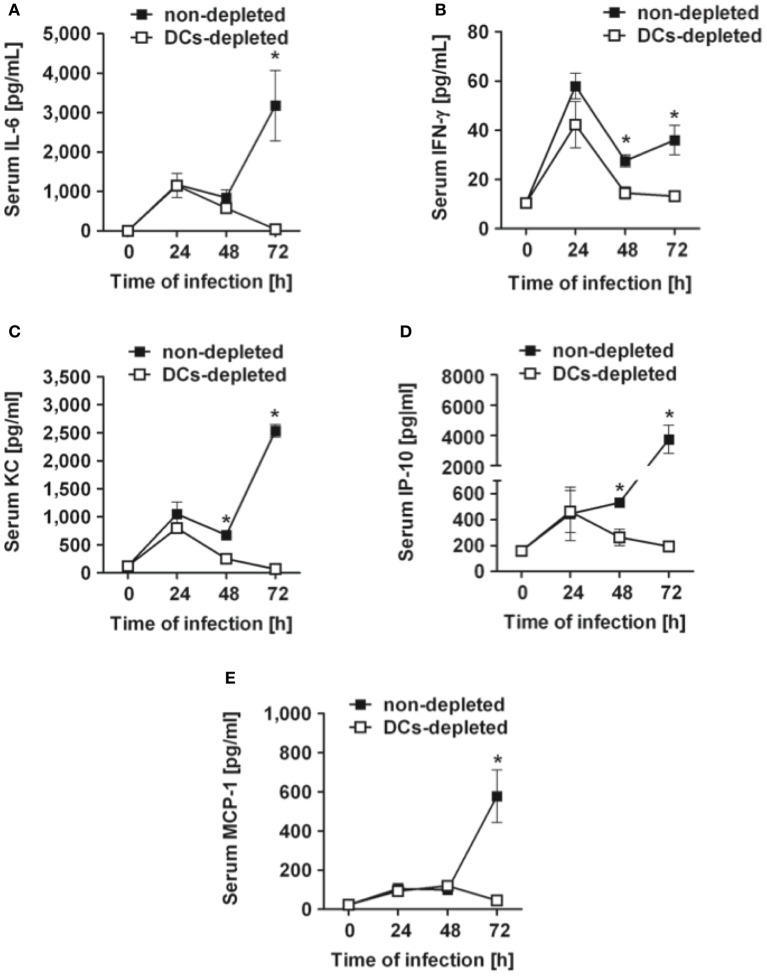
**Kinetics of serum cytokines and chemokines in DCs-depleted and non-depleted mice during the course of *S. pneumoniae* infection**. DCs-depleted (white symbols) and non-depleted (black symbols) CD11c-DTR chimera mice were challenged intranasally with 1 × 10^8^ CFU of *S. pneumoniae* D39 and the levels of IL-6 **(A)**, IFN-γ **(B)**, KC **(C)**, IP-10 **(D)**, and MCP-1 **(E)** in serum were determined by multiplex technology at 24, 48, and 72 h after bacterial inoculation. Each symbol represents the mean ± *SD* of the compilation of three independent experiments. ^*^p < 0.05.

Determination of serum cytokines in DCs-depleted and non-depleted mice at progressing times after bacterial inoculation demonstrated that the systemic inflammatory response triggered by *S. pneumoniae* infection was also much more tempered in the absence of DCs. Thus, significantly lower levels of circulating serum IL-6 (Figure 3A), IFN-γ (Figure 3B), KC (Figure 3C), IP-10 (Figure 3D) and MCP-1 (Figure 3E) were detected at 72 h of infection in DCs-depleted than in non-depleted mice. These results indicate that depletion of DCs results in attenuated systemic inflammation in *S. pneumoniae*-infected mice.

### Depletion of DCs does not affect the recruitment of leukocytes into the *S. pneumoniae*-infected lungs

**Figure 4:**
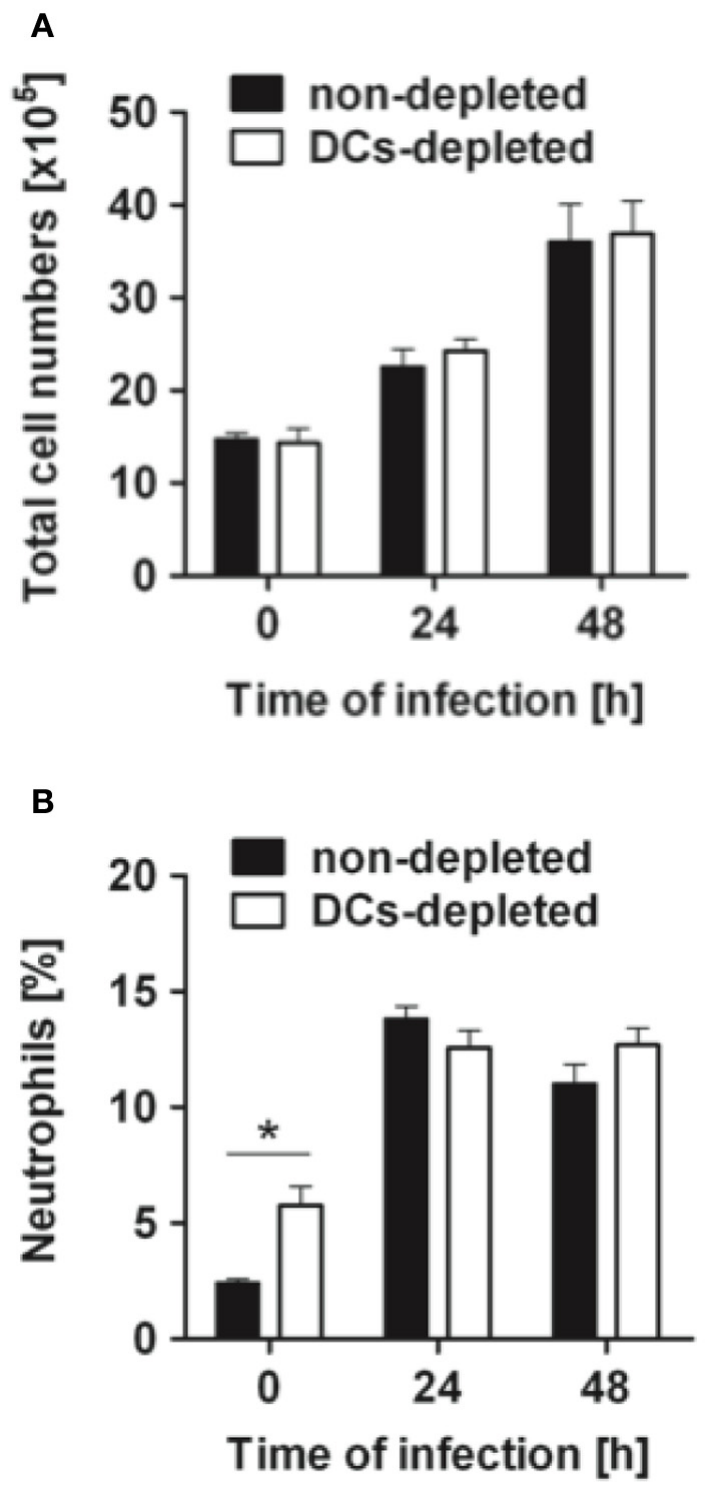
**Cell recruitment in the lungs of DCs-depleted and non-depleted mice during pneumococcal pneumonia**. DCs-depleted (white bars) and non-depleted (black bars) CD11c-DTR chimera mice were intranasally infected with 1 × 10^8^ CFU of *S. pneumoniae* D39, the lungs taken before (0 h) or at 24 and 48 h after bacterial inoculation and dispersed with collagenase and DNase to obtain a single cell suspension. Cells were counted **(A)** and then stained with antibodies against Gr-1 to identify the neutrophil population **(B)** by flow cytometry analysis. Each bar represents the mean ± *SD* of triplicate samples. One representative experiment out of three is shown. ^*^p < 0.05.

Given that DCs depletion improved the host resistance to *S. pneumoniae*, we investigated whether the superior resistance of DCs-depleted mice was due to more efficient recruitment of cells involved in bacterial clearance into the lungs. DCs-depleted and non-depleted CD11c-DTR chimera mice were intranasally infected with *S. pneumoniae* and the total amount of cells in the lungs was determined before and at 24 h and 48 h after bacterial inoculation. As shown in Figure 4A, a comparable time-dependent increase in the total amount of cells recruited into the infected lungs was observed in both DCs-depleted and non-depleted mice. The recruitment of neutrophils (Figure 4B) or CD4+ T cells (data not shown) was similar between both groups at the different times of infection.

**Figure 5:**
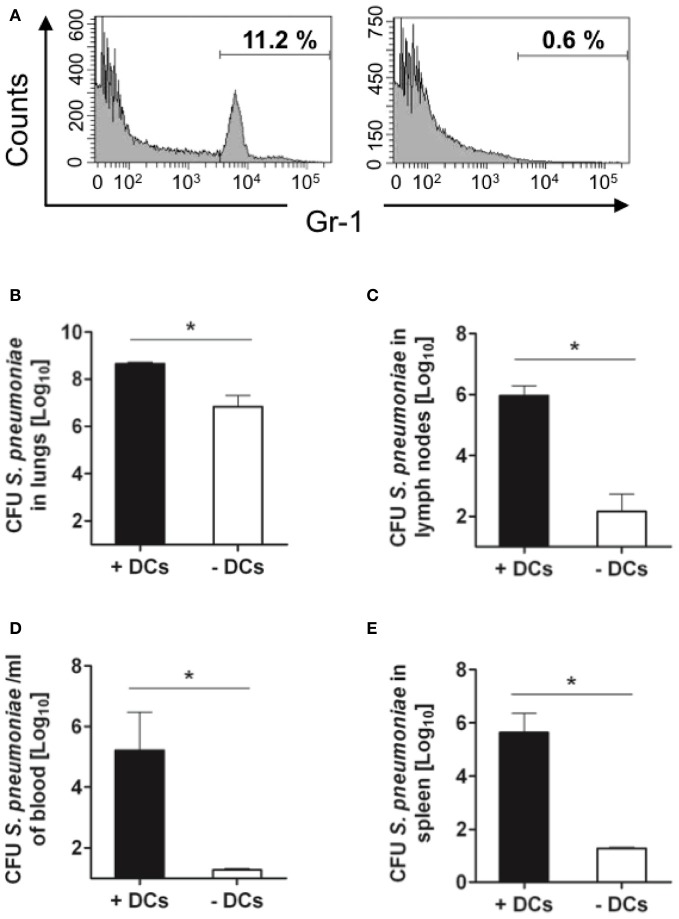
**Effect of neutrophil depletion in control of *S. pneumoniae* by DCs-depleted and non-depleted mice**. DT-treated (white bars) and untreated (black bars) CD11c-DTR chimera mice were depleted of neutrophils and intranasally infected with 1 × 10^8^ CFU of *S. pneumoniae* D39. **(A)** Histograms showing the percentage of neutrophils in blood before (left histogram) and after (right histogram) depletion. Bacterial loads in the lungs **(B)**, mediastinal lymph nodes **(C)**, blood **(D)**, and spleen **(E)** were determined at 48 h of infection. Each symbol represents an individual mouse. One representative experiment out of three is shown. ^*^p < 0.05.

Interestingly, depletion of DCs after treatment with DT induced a significant increase in the accumulation of neutrophils within the lungs of treated mice (Figures 1B, 4B). This phenomenon associated with DT treatment has been previously described by Tittel et al. (2012). As neutrophils are the most important innate immune cells for early defense against *S. pneumoniae* (Matthias et al., 2008; Standish and Weiser, 2009), it can be hypothesized that a higher amount of neutrophils present in the lungs of DT-treated mice previous to bacterial challenge can be responsible for the lower bacterial burdens observed in these animals at later time points. However, the fact that the amount of bacteria in the lungs at 24 h after intranasal challenge is comparable in PBS- and DT-treated mice gives clear indication that the greater amount of neutrophils in the lungs of the later mice is not responsible for the superior resistance of these animals. To further demonstrate this argument, DT-treated (DCs-depleted) or PBS-treated (non-depleted) CD11c-DTR chimera mice were depleted of neutrophils and subsequently challenged intranasally with *S. pneumoniae*. The efficacy of neutrophil depletion was >95% as shown in Figure 5A. The pneumococcal burdens were assessed in different tissues at 48 h of infection. If the superior number of neutrophils present in the lungs of DCs-depleted mice would be responsible for the lower bacterial loads detected in the organs of these animals, an equal amount of bacteria could be expected in the organs of DCs-depleted and non-depleted mice after neutrophil ablation. As shown in Figure 5, the amount of bacteria in the lungs (Figure 5B), lymph nodes (Figure 5C), blood (Figure 5D), and spleen (Figure 5E) was significantly lower in DCs-depleted than in non-depleted mice in the absence of neutrophils. These results indicate that the beneficial effect conferred by depletion of DCs during pneumococcal infection was not the consequence of the increased number of neutrophils associated with the depletion procedure.

### *S. pneumoniae* disseminates from the lungs to the mediastinal lymph nodes in a cell-independent manner

**Figure 6:**
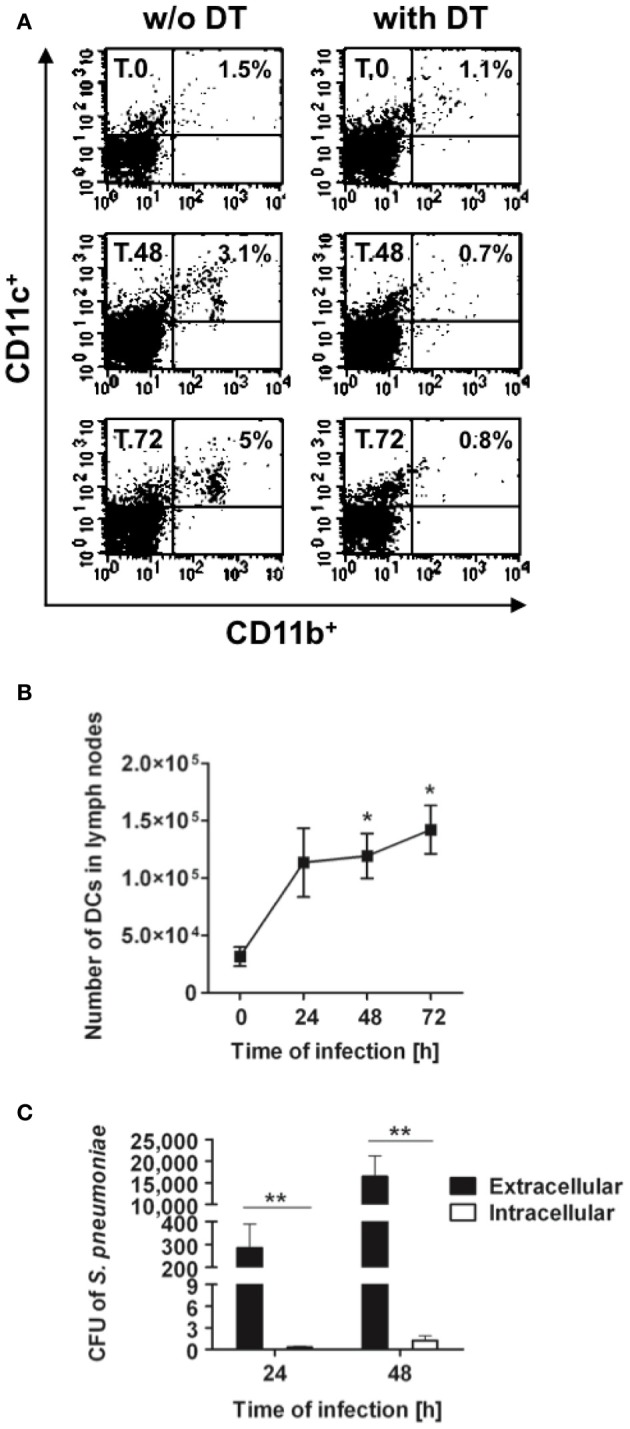
**Trafficking of DCs to the mediastinal lymph nodes after intranasal infection with *S. pneumoniae***. DCs-depleted and non-depleted CD11c-DTR chimera mice were intranasally infected with 1 × 10^8^ CFU of *S. pneumoniae*, the mediastinal lymph nodes were removed before (0 h) and at 24, 48, and 72 h after bacterial inoculation and transformed in a single cell suspension. Cells were then counted, stained with anti-mouse CD11c and anti-mouse CD11b antibodies and analyzed by flow cytometry. **(A)** Representative dot plots of DCs in the mediastinal lymph nodes of *S. pneumoniae*-infected DCs-depleted (right histograms) and non-depleted (left histograms) mice. The numbers in each dot plot represent the percentage of DCs. One representative experiment out of three is shown. **(B)** Total amount of DCs in the mediastinal lymph nodes of *S. pneumoniae*-infected DCs-depleted (white symbols) and non-depleted (black symbols) CD11c-DTR chimera mice. Each symbol represents the mean ± *SD* of three independent experiments. **(C)** Quantification of viable intracellular (white bars) and extracellular (black bars) *S. pneumoniae* in the mediastinal lymph nodes of *S. pneumoniae*-infected mice at 24 and 48 h after bacterial inoculation. Each bar represents the mean ± *SD* of triplicate samples. One representative experiment out of three is shown. ^*^p < 0.05 and ^**^p < 0.01.

**Figure 7:**
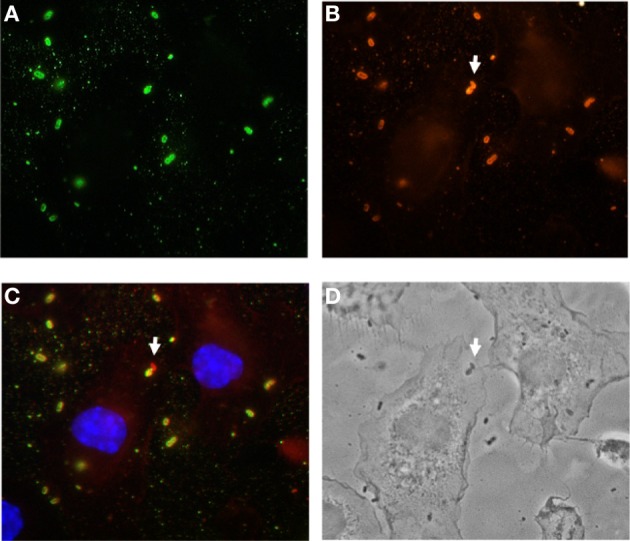
**Poor uptake of *S. pneumoniae* by bone marrow-derived DCs in *in vitro* assays**. Bone marrow-derived DCs were infected with *S. pneumoniae* for 2 h, fixed, and stained for with polyclonal rabbit anti-*S. pneumoniae* antibodies, followed by Alexa green-conjugated goat anti-rabbit antibodies **(A)**. Cells were then permeabilized with 0.025% Triton X-100 in PBS, and intracellular bacteria were stained by anti-*S. pneumoniae* antibodies, followed by Alexa red-conjugated goat anti-rabbit antibodies **(B)**. In the merged image shown in **(C)**, extracellular bacteria are yellow-green, intracellular bacteria are red and the DNA in the nucleus of DCs is stained in blue. The white arrow indicated an intracellular *S. pneumoniae*. **(D)** Phase contrast picture showing the contour of the DCs.

As DCs-depleted mice exhibited a better capacity to restrict *S. pneumoniae* extrapulmonary dissemination, we next investigated the potential mechanism by which DCs facilitated *S. pneumoniae* dissemination from the lungs. Evidence has been provided that DCs can be used by certain pathogens as a “Trojan horse” or vehicle to disseminate systemically (Pron et al., 2001; Wu and KewalRamani, 2006; Bierly et al., 2008; Wykes and Horne-Debets, 2012), we examined whether this could also be the case for *S. pneumoniae*. Results in Figures 6A,B revealed a time-dependent increase in the amount of DCs migrating in the draining lymphoid tissue after intranasal inoculation with *S. pneumoniae*. However, the results of a gentamicin protection assay indicated that the main fraction of the bacterial population within the lymph nodes was located extra- rather than intracellularly within the DCs or other cell type (Figure 6C). Additionally, we determined the ability of bone marrow-derived murine DCs to phagocyte *S. pneumoniae* in *in vitro* assays. DCs were incubated with *S. pneumoniae* at a MOI of 20:1 for 2 h, unbound bacteria were removed by washing and DCs were stained for immune fluorescence examination. To differentiate internalized from adherent extracellular bacteria we used double immunofluorescence analysis with intracellular bacteria appearing red, while externally-associated bacteria appear yellow-green and the nuclei of DCs are stained in blue (Figure 7). As seen in Figure 7C, the immunofluorescence microscopy examination of infected DCs further confirmed the low proficiency of DCs to phagocyte *S. pneumoniae.* Increased incubation time or multiplicity of infection did not improve the frequency of intracellular bacteria but rather compromised the viability of the DCs (data not shown).

Together, these results suggest that *S. pneumoniae* disseminated from the lungs into the regional lymph nodes in a cell-independent manner and that this mode of dissemination was much more efficient in the presence of DCs.

### *S. pneumoniae* induces expression of MMP-9 in DCs

**Figure 8:**
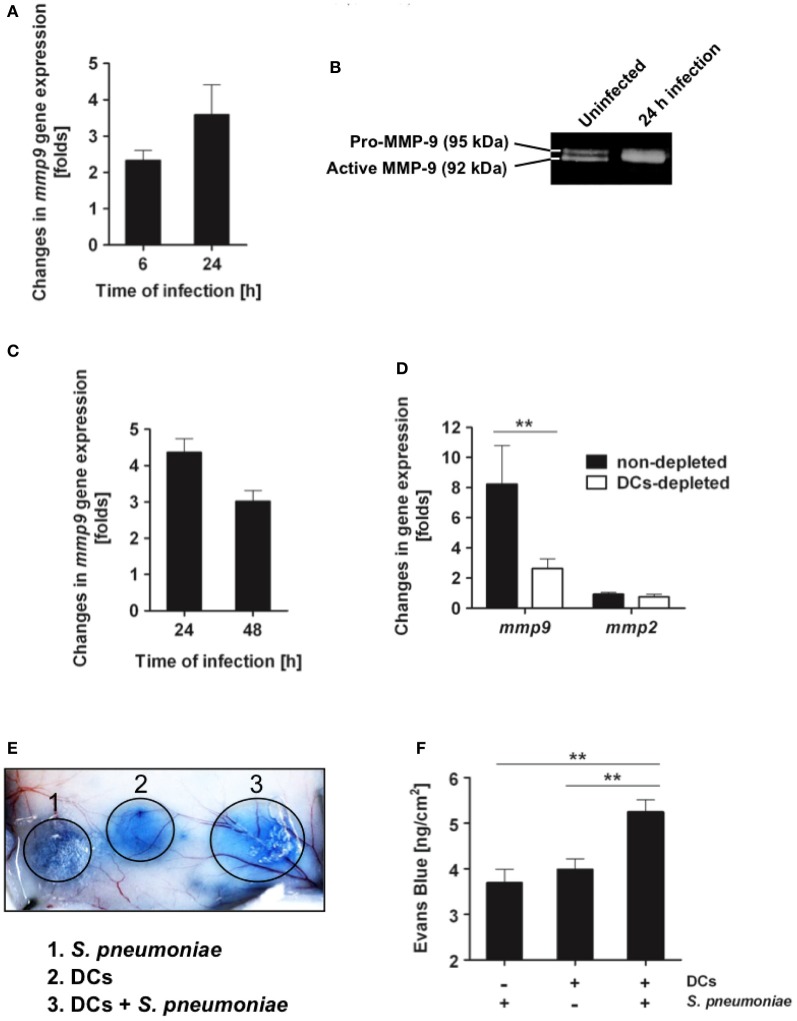
***S. pneumoniae* triggers production of MMP-9 on *in vitro* cultured DCs and in lung tissue during *in vivo* infection. (A)** Bone marrow-derived DCs were infected with *S. pneumoniae* (MOI 20:1), RNA was extracted from DCs at 6 and 24 h postinfection and subjected to real-time PCR for quantification of *mmp-9* gene expression. Results are expressed as fold-change in MMP-9 mRNA of infected DCs over the amount of MMP-9 mRNA in uninfected DCs. Each bar represents the mean of three independent experiments. **(B)** Representative gelatin zymography demonstrating the up-regulation of the active form of MMP-9 in the supernatant of *S. pneumoniae*-infected DCs at 24 h postinfection. **(C)** Up-regulation of *mmp-9* gene expression in the lungs of BALB/c mice during infection with *S. pneumoniae*. BALB/c mice were intranasally infected with 1 × 10^8^ CFU of *S. pneumoniae* D39, the RNA was isolated from the lungs at 24 and 48 h of infection and subjected to real-time PCR for detection of MMP-9 mRNA. Each bar represents the mean value of three independent experiments. **(D)** Up-regulation of *mmp-9* gene expression in the lungs of DCs-depleted (white bar) or non-depleted (black bar) CD11c-DTR chimera mice at 24 h of infection with 1 × 10^8^ CFU of *S. pneumoniae* D39. Each bar represents the mean value of three independent experiments. **(E)** Vascular permeability induced by the supernatant from either *S. pneumoniae* (1), DCs (2), or *S. pneumoniae*-infected DCs (24 h postinfection) (3). Supernatant (50 μl) was intradermally applied into the skin of Evans blue-treated mice and vascular leakage of Evans blue was visualized 1 h thereafter. A representative experiment out of three is shown. **(F)** Quantification of Evans blue leakage in the skin of mice after application of supernatant from *S. pneumoniae*, DCs or *S. pneumoniae*-infected DCs (24 h postinfection). Each bar represents the mean ± *SD* of three independent experiments. ^**^p < 0.01.

After encountering pathogens in peripheral tissue, immature DCs recognize microbial components, undergo maturation and migrate to the regional lymph nodes (Banchereau et al., 2000). Trafficking of DCs to lymph nodes involves several steps including the expression of chemokines receptors that regulate DC chemotaxis through chemokines gradient as well as transit through connective tissues and cross basement membranes, which mainly consist of laminin, type IV collagen, and heparan sulfate proteoglycans (Alvarez et al., 2008). The metalloproteinases MMP-2 and MMP-9 in particular are especially important in DCs migration, since they cleave collagen IV, a major component of basement membranes (Ratzinger et al., 2002; Vermaelen et al., 2003; Chiyasu et al., 2004). Several pathogens have been reported to induce the production and activation of MMP-9 by host cells to open the way through tissue and thereby to facilitate dissemination within the host (Luplertlop et al., 2006; Ramu et al., 2008; Nishikaku et al., 2009; Marsac et al., 2011). Therefore, we next investigated whether *S. pneumoniae* might exploit the DC-derived proteolysis to enhance its dissemination from the lungs to the regional lymph nodes. To this end, we analyzed the expression and activity of MMP-9 in DCs after exposure to *S. pneumoniae.* The results showed that *S. pneumoniae* induced *mmp-9* gene expression in cultured bone marrow-derived DCs (Figure 8A). *S. pneumoniae* also stimulated the activation of MMP-9 released by *S. pneumoniae*-infected DCs to its active isoform as demonstrated by the gelatinolytic activity of the culture supernatant (Figure 8B). MMP-9 was highly up-regulated in the lungs of mice after intranasal challenge with *S. pneumoniae* (Figure 8C) and depletion of DCs resulted in significant reduction of MMP-9 levels in the infected lungs (Figure 8D). MMP-2, an additional metalloproteinase reported to be involved in DCs migration (Ratzinger et al., 2002), was induced to a lesser extent in the murine infected lungs (Figure 8D). However, in contrast to MMP-9, the level of MMP-2 was not affected by the depletion of DCs (Figure 8D). These results suggest that DCs are largely involved in the production of MMP-9 during pneumococcal pneumonia.

Furthermore, functional analysis using an *in vivo* vascular permeability assay revealed that supernatants from DCs infected with *S. pneumoniae* had increased basement membrane permeability than those from uninfected DCs (Figures 8E,F). Together, these results indicate that *S. pneumoniae* enhances the capacity of DCs to degrade extracellular matrices and the permeability of the basement membranes.

## Discussion

The results of this study demonstrate that *in vivo* depletion of DCs enhanced the resistance of mice to intranasal challenge with *S. pneumoniae.* These results are in line with an earlier study showing that increased numbers of DCs in the lungs of mice after treatment with FMS-like tyrosine kinase 3 ligand (Flt3L), which is a hemopoietic growth factor required for DC development in peripheral lymphoid tissue (Liu and Nussenzweig, 2010), significantly increased mortality and morbidity of mice challenged intranasally with pneumococci (Winter et al., 2007). The improved control of *S. pneumoniae* infection after DC depletion observed in our study was neither mediated by an increased recruitment of cells involved in bacterial clearance at the site of infection nor by increased bactericidal capacity of neutrophils in the absence of DCs as recently reported in an experimental mouse model of *Yersinia enterocolitica* (Autenrieth et al., 2012). The superior resistance of DCs-depleted mice to *S. pneumoniae* seems to be related to a better capacity to restrict bacterial dissemination from the local site of infection. Hence, we speculate that the higher bacterial loads observed in the lungs of non-depleted animals at 48 h and 72 h of infection in comparison with DCs-depleted animals are due to bacterial entrapment in the pulmonary capillaries after been released into the bloodstream from heavily infected peripheral organs rather than to an inferior capacity of these animal to eliminate pneumococci.

It has been reported that treatment of CD11c-DTR mice with DT can also result in the depletion of CD11c+ alveolar macrophages (van Rijt et al., 2005). However, previous studies using intranasal instillation of liposomal dichloromethylene-bisphosphonate to achieve specific depletion of alveolar macrophages demonstrated that depletion of alveolar macrophages did not affect the bacterial burdens in the lungs and blood of mice after intranasal infection with *S. pneumoniae* (Knapp et al., 2003). Therefore, the benefit conferred by the depletion of CD11c+ cells on the course of respiratory *S. pneumoniae* infection observed in our study can be attributed to the depletion of DCs rather than to the depletion of alveolar macrophages.

To migrate from the lungs to the draining lymph nodes, *S. pneumoniae* must traverse tissue barriers such as the epithelium, basal membranes and endothelium. Evidence is accumulating that certain bacterial pathogens can take advantage of the migratory capacity of DCs and use them as a “Trojan horse” or vehicle to disseminate systemically (Pron et al., 2001; Wu and KewalRamani, 2006; Bierly et al., 2008; Wykes and Horne-Debets, 2012). This led us to search for an association between DCs and *S. pneumoniae* in the infected lymph nodes. However, we found that pneumococci were mostly located extracellularly, implying that *S. pneumoniae* disseminated from the lungs into the regional lymph nodes in a cell-free manner. In this regard, proteolytic cleavage of the extracellular matrix and basement membranes is a prerequisite for *S. pneumoniae* to travel from lungs to lymph nodes. Invasive pathogens such as *Pseudomonas aeruginosa* and Clostridium spp. secrete proteases that lead to tissue damage and thereby enhance bacterial invasiveness (Singh et al., 2012). On the other hand, a number of invasive bacteria including *S. pneumoniae* produce low levels of proteases and therefore they use a different mechanism for the degradation of extracellular matrix during tissue penetration. One of these mechanisms relies on the interaction with the host protease-dependent pathways such as fibrinolysis, coagulation, and complement activation (Bhattacharya et al., 2012). In this regard, *S. pneumoniae* expresses surface receptors which capture host plasminogen and thereby generate and utilize host-derived plasmin activity to degrade laminin and other glycoproteins in the extracellular matrix and basement membranes (Eberhard et al., 1999; Bergmann et al., 2005; Bergmann and Hammerschmidt, 2007). The plasminogen-plasmin receptors at the surface of *S. pneumoniae* include the α-enolase (Bergmann et al., 2001), the glyceraldehyde-3-phosphate dehydrogenase (GAPDH) (Bergmann et al., 2004) and the surface-exposed choline-binding protein E (CBPE, also referred to as Pce) (Attali et al., 2008). Plasmin, however, is not very efficient in collagen breakdown, whereas the matrix metalloproteinases (MMPs) have the ability to degrade collagens, which are the major components responsible for the barrier function of the extracellular matrix (Hotary et al., 2000). Notably, we found that MMP-9 activity was up-regulated in DCs after exposure to *S. pneumoniae*.

The process of DC migration to regional lymph nodes is rather complex and involves multiple steps each orchestrated by specific factors. These include chemo-attractants to dictate the direction of migration (Sallusto et al., 1999; Sozzani et al., 1999), adhesion molecules acting as docking stations (D'Amico et al., 1998), and proteinases to break physical barriers and to open the way in the extracellular matrix (D'Amico et al., 1998; Sozzani et al., 1999). Both metalloproteinase MMP-2 and MMP-9 degrade collagen IV and act as major players in DC migration. In particular, the role of MMP-9 in DC migration has been demonstrated *in vitro*, in skin explant models, and *in vivo* (Hollender et al., 2002; Ratzinger et al., 2002; Ichiyasu et al., 2004; Chabot et al., 2006; Zozulya et al., 2007). We found that MMP-9 was highly up-regulated in the lungs of mice after intranasal challenge with *S. pneumoniae.* Furthermore, depletion of DCs resulted in significant reduction in the levels of MMP-9 in the infected lungs, which supports that DCs are either a direct source of MMP-9 during pneumococcal pneumonia or that they modulate MMP-9 production by other cell types. Thus, it is tempting to speculate that the increased production of MMP-9 by DCs during pneumococcal pneumonia and the resulting breakdown of extracellular matrix and basal membranes on their way to the regional lymph nodes can open tissue barriers and thereby facilitate the extrapulmonary dissemination of *S. pneumoniae*. In agreement with our results, Yasuda et al. (2010) reported enhanced airway resistance to *S. pneumoniae* associated with suppression of MMP-9 expression/activation in mice. Furthermore, studies performed in a rat model of pneumococcal meningitis documented induction of MMPs in brain parenchymal tissue and significantly reduced brain injury after MMP chemical inhibition (Leib et al., 2000, 2001). Elevated blood and bronchoalveolar lavage levels of different MMPs have also been reported in patients with pneumonia (Hartog et al., 2003; Yang et al., 2005). More interestingly, high MMPs levels in these patients were related to increased clinical severity (Hartog et al., 2003). The MMPs are produced as inactive precursors and a specific activation process in the extracellular milieu is required for expression of their proteolytic activity against extracellular matrix proteins (Ra and Parks, 2007). In this context, *S. pneumoniae* has also been reported to produce or recruit proteases that activate pro-MMPs (Oggioni et al., 2003).

Murine DCs are an unquestionably valuable tool to investigate the interactions of DCs with pathogens. Although differences in the production of cytokines have been reported between human and murine DCs after *in vitro* infection with *S. pneumoniae* (Littmann et al., 2009), the overall performance of murine DCs during *in vivo* infection can be relevant to human DCs since, like human DCs, murine DCs express receptors for recognition of bacterial components, they express MHC class II molecules and costimulatory molecules involved in antigen presentation, and they mature and migrate in response to danger signals (Banchereau et al., 2000). Therefore, despite its limitations, murine DCs remain one of the most important experimental systems to investigate the role of DCs during infection and experiments in mice have been pivotal to clarify the central role of DCs in the immune function. Studies addressing the *in vivo* function of DCs in human are scarce. Two genetically defined syndromes of DC deficiency have recently been described in humans (Bigley et al., 2011; Collin et al., 2011; Hambleton et al., 2011). Mutation of GATA-binding factor 2 (*GATA2*) results in absence of monocytes and DCs (Hambleton et al., 2011) and mutations in the gene encoding the interferon regulatory factor 8 (*IRF8*) were found to compromise DC development in the affected patients (Collin et al., 2011; Hambleton et al., 2011). The phenotype in both cases includes susceptibility to infection with *Mycobacterium* spp. (Bigley et al., 2011; Collin et al., 2011; Salem and Gros, 2013). Intriguingly, in any of the cases the patients exhibited increased susceptibility to *S. pneumoniae* or other extracellular pathogen. An impaired production of IL-12 and IFN-γ due to the absence of monocytes and DCs could partly explain why these patients exhibit an increased susceptibility to poorly virulent strains of *Mycobacterium* spp (Collin et al., 2011). While these cytokines are essential for the control of mycobacterial infections (Jouanguy et al., 1999), they play a minor role in host defense against *S. pneumoniae* (Lauw et al., 2002). This can provide a plausible explanation for the lack of susceptibility of DC-deficient patients to pneumococcal infections.

The pathogenesis of the experimental murine pneumococcal pneumonia described in this study can be divided into different phases: during the very early phase of infection and shortly after bacterial inoculation, pneumococci will encounter the resident alveolar macrophages, which will try to phagocyte and kill the incoming bacteria. *S. pneumoniae* is capable to survive at this stage by expressing a thick polysaccharide capsule that interferes with the phagocytic activity of the alveolar macrophages. The next phase of infection (24 h) is characterized by a massive influx of neutrophils and bacterial dissemination from the lungs to the regional lymph nodes. Dissemination of *S. pneumoniae* at this stage of infection parallels the migration of DCs from the lungs to draining lymph nodes. Based on the observations of our study, it can be speculated that pneumococci take advantage of the ability of the DCs to open tissue barriers on their way to the lymph nodes to promote their own dissemination from the local site of infection. For this reason, the amount of bacteria reaching the lymph nodes will be much lower in the absence of DCs. Bacterial colonization of the lymph nodes (24 h of infection) precedes the appearance of the pathogen in the blood and the spreading to distant organs where the microorganisms proliferate and establish new focus of infection (48 h of infection). Due to the lower amount of bacteria reaching the lymph nodes in DCs-depleted mice, the amount of pneumococci spreading from the lymph nodes into the bloodstream and systemic organs will also be lower in mice depleted of DCs. A fraction of the progressively increasing bacterial burden will be discharged from the infected organs into the bloodstream and thereby transported to the lungs. Hence, the higher bacterial loads detected in the lungs of non-depleted mice at 48 h and 72 h of infection might be the results of a larger amount of bacteria discharged into the bloodstream from the more heavily infected systemic organs.

Taken together, our results suggest that modulation of DCs during pneumococcal pneumonia might provide an interesting means to prevent or delay extrapulmonary bacterial dissemination.

### Conflict of interest statement

The authors declare that the research was conducted in the absence of any commercial or financial relationships that could be construed as a potential conflict of interest.
