# Supplementary material for: Lung dendritic cells facilitate extrapulmonary bacterial dissemination during pneumococcal pneumonia
Source: Front Cell Infect Microbiol. 2013 Jun 21;3:21. doi: 10.3389/fcimb.2013.00021 (PMC3689026; doi:10.3389/fcimb.2013.00021)
Supplement: Supplementary file 1 [file DataSheet1.PDF]

Supplementary Figure S1

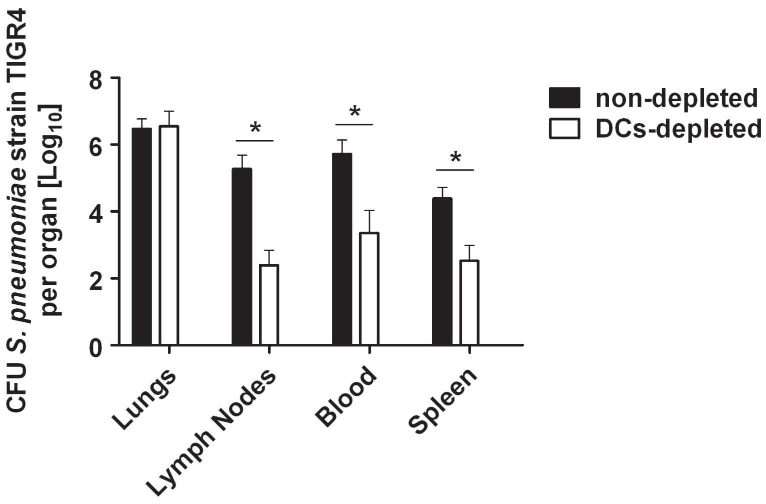

Supplementary Figure S1. Bacterial burdens in the organs of DC-depleted and non-depleted mice after respiratory challenge with *S. pneumoniae* strain TIGR4. DC-depleted (white bars) and non-depleted (black bars) CD11c-DTR chimera mice were intranasally inoculated with 1 x 10^7 CFU of *S. pneumoniae* strain TIGR4 and the bacterial loads determined in the lungs, mediastinal lymph nodes, blood and spleen at 24 h after bacterial inoculation. Each symbol represents an individual animal. One experiment out of three is shown. *, $p < 0.05$.

Supplementary Figure 2

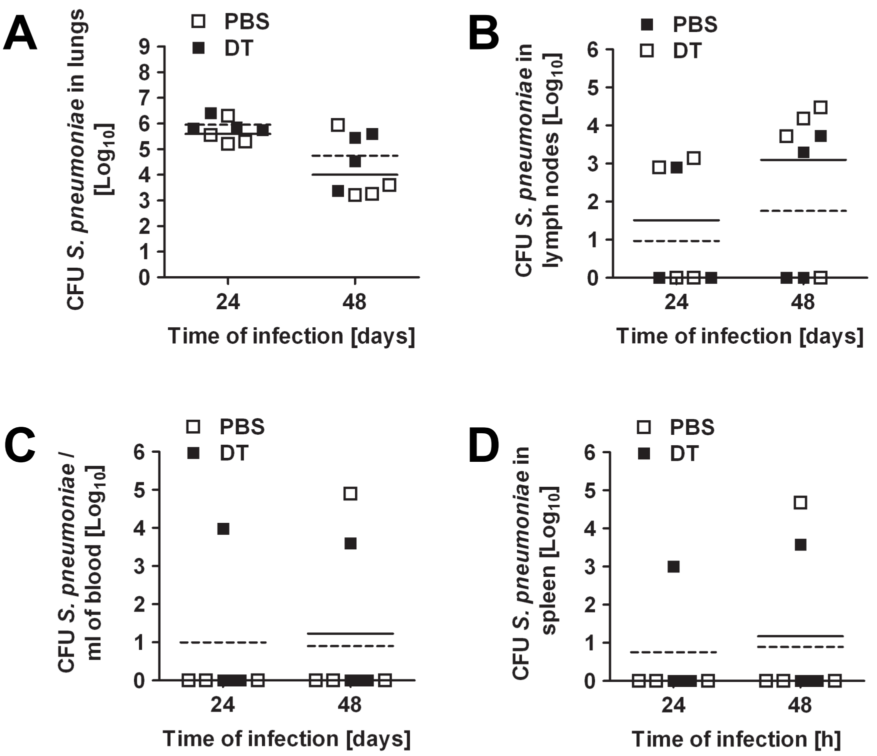

Supplementary Figure S2. Treatment with DT does not affect the course of *S. pneumoniae* respiratory infection in BALB/c mice. BALB/c mice were treated with a daily intraperitoneal injection of DT in PBS (8 ng/g body weight) or with PBS alone starting 2 days before infection. DT-treated (black symbols) and PBS-treated (white symbols) BALB/c mice were intranasally inoculated with 1 x 10^8 CFU of *S. pneumoniae* D39 and the bacterial loads determined in the lungs (A), mediastinal lymph nodes (B), blood (C) and spleen (D) at 24 and 48 h after bacterial inoculation. Each symbol represents an individual animal. Horizontal continuous lines indicate the mean value of PBS-treated mice and horizontal broken lines indicate the mean value of DT-treated mice. One experiment out of three is shown.
